# Risk Factors Associated With COVID-19 Transmission Among US Air Force Trainees in a Congregant Setting

**DOI:** 10.1001/jamanetworkopen.2021.0202

**Published:** 2021-02-25

**Authors:** Joseph E. Marcus, Dianne N. Frankel, Mary T. Pawlak, Theresa M. Casey, Robert J. Cybulski, Erin Enriquez, Jason F. Okulicz, Heather C. Yun

**Affiliations:** 1 Infectious Diseases Service, Brooke Army Medical Center, Joint Base San Antonio, Texas; 2 Trainee Health Surveillance, 559th Medical Group, Joint Base San Antonio–Lackland, Texas; 3 Clinical Microbiology, Department of Pathology and Area Laboratory Services, Brooke Army Medical Center, Joint Base San Antonio, Texas

## Abstract

**Importance:**

Owing to concerns of coronavirus disease 2019 (COVID-19) outbreaks, many congregant settings are forced to close when cases are detected because there are few data on the risk of different markers of transmission within groups.

**Objective:**

To determine whether symptoms and laboratory results on the first day of COVID-19 diagnosis are associated with development of a case cluster in a congregant setting.

**Design, Setting, and Participants:**

This cohort study of trainees with COVID-19 from May 11 through August 24, 2020, was conducted at Joint Base San Antonio–Lackland, the primary site of entry for enlistment in the US Air Force. Symptoms and duration, known contacts, and cycle threshold for trainees diagnosed by reverse transcription–polymerase chain reaction were collected. A cycle threshold value represents the number of nucleic acid amplification cycles that occur before a specimen containing the target material generates a signal greater than the predetermined threshold that defines positivity. Cohorts with 5 or more individuals with COVID-19 infection were defined as clusters. Participants included 10 613 trainees divided into 263 parallel cohorts of 30 to 50 people arriving weekly for 7 weeks of training.

**Exposures:**

All trainees were quarantined for 14 days on arrival. Testing was performed on arrival, on day 14, and anytime during training when indicated. Protective measures included universal masking, physical distancing, and rapid isolation of trainees with COVID-19.

**Main Outcomes and Measures:**

Association between days of symptoms, specific symptoms, number of symptoms, or cycle threshold values of individuals diagnosed with COVID-19 via reverse transcription–polymerase chain reaction and subsequent transmission within cohorts.

**Results:**

In this cohort study of 10 613 US Air Force basic trainees in 263 cohorts, 403 trainees (3%) received a diagnosis of COVID-19 in 129 cohorts (49%). Among trainees with COVID-19 infection, 318 (79%) were men, and the median (interquartile range [IQR]) age was 20 (19-23) years; 204 (51%) were symptomatic, and 199 (49%) were asymptomatic. Median (IQR) cycle threshold values were lower in symptomatic trainees compared with asymptomatic trainees (21.2 [18.4-27.60] vs 34.8 [29.3-37.4]; *P* < .001). Cohorts with clusters of individuals with COVID-19 infection were predominantly men (204 cohorts [89%] vs 114 cohorts [64%]; *P* < .001), had more symptomatic trainees (146 cohorts [64%] vs 53 cohorts [30%]; *P* < .001), and had more median (IQR) symptoms per patient (3 [2-5] vs 1 [1-2]; *P* < .001) compared with cohorts without clusters. Within cohorts, subsequent development of clusters of 5 or more individuals with COVID-19 infection compared with those that did not develop clusters was associated with cohorts that had more symptomatic trainees (31 of 58 trainees [53%] vs 43 of 151 trainees [28%]; *P* = .001) and lower median (IQR) cycle threshold values (22.3 [18.4-27.3] vs 35.3 [26.5-37.8]; *P* < .001).

**Conclusions and Relevance:**

In this cohort study of US Air Force trainees living in a congregant setting during the COVID-19 pandemic, higher numbers of symptoms and lower cycle threshold values were associated with subsequent development of clusters of individuals with COVID-19 infection. These values may be useful if validated in future studies.

## Key Points

**Question:**

In congregant settings with the introduction of coronavirus disease 2019 (COVID-19), what clinical and laboratory findings are associated with an outbreak?

**Findings:**

In this cohort study of 10 613 US Air Force basic trainees living congregantly in 263 cohorts, 3% were diagnosed with COVID-19 infection. Cohorts with trainees with more symptoms and lower cycle threshold values on reverse transcription–polymerase chain reaction assay were significantly associated with greater risk of transmission of COVID-19 within their cohorts.

**Meaning:**

In this study, a higher number of symptoms and lower cycle threshold values were associated with subsequent clusters of outbreaks within cohorts and may be useful as risk factor measures if validated in future studies.

## Teaser

This cohort study examines whether symptoms and laboratory results on the first day of COVID-19 diagnosis are associated with development of a case cluster in a congregant setting.

## Introduction

Coronavirus disease 2019 (COVID-19) is a syndrome caused by infection with severe acute respiratory syndrome coronavirus 2 (SARS-CoV-2) and associated with outbreaks in congregant settings.^1,2,3^ Despite its rapid worldwide emergence, there have been reports of effective implementation of nonpharmaceutical interventions, such as physical distancing and rapid isolation of symptomatic patients, that have prevented transmission in both institutions and the general population.^4,5,6^

Reports characterizing risk factors for COVID-19 spread have suggested that time with infected individuals is a major driver for transmission.^7^ In a study evaluating secondary infection in China, more severe disease in the source patient was additionally noted as a risk factor for disease dissemination.^8^ Many of these studies have occurred in the setting of no or evolving nonpharmaceutical interventions, which makes it challenging to interpret the contributions of public health measures compared with patients in disease spread.

Adding to the uncertainty, the role of asymptomatic transmission in patients with COVID-19 has not been clearly defined. Although there are many reports of asymptomatic disease acquisition,^9,10,11^ its actual contribution to outbreaks has been difficult to elucidate. Modeling studies have estimated that to stop spread, there needs to be rapid isolation of symptomatic patients as well as interventions to minimize asymptomatic transmission, such as universal masking and physical distancing.^12^ These studies are limited by assumptions but provide the framework for the current COVID-19 response.

One value that has potential to be associated with risk for an outbreak of COVID-19 is reverse transcription–polymerase chain reaction (RT-PCR) cycle threshold (Ct). A Ct value represents the number of nucleic acid amplification cycles that occur before a specimen containing the target material generates a signal greater than the predetermined threshold that defines positivity. Ct values inversely correlate with the amount of target material in a tested specimen and may correlate with the infectious load of the assay’s target. In the case of SARS-CoV-2, Ct values may be correlated with outcomes in patients.^13,14,15^ Ct values have not been compared between cohorts to determine subsequent risk of clustered cases. There have been criticisms of Ct value when patients are compared, resulting from the challenges of normalizing Ct values generated on different testing platforms and a range of preanalytic factors, including specimen collection.^16^

Basic military training is the first step in the transition of a civilian to an enlisted member of the US Air Force. It brings together more than 39 000 trainees every year from around the United States. Trainees arrive weekly and are immediately divided by sex in parallel cohorts of 30 to 50 members. These cohorts require trainees to be in close proximity through all of their daily training as well as their free time in open-bay dormitories. The median age for trainees is 20 years, and approximately 75% of entering trainees are men. Basic military training has been associated with viral outbreaks in recent years, including adenovirus^17,18^ and coxsackievirus,^19^ and a strong public health infrastructure was in place before COVID-19 for monitoring emerging infections.

In March and April 2020, among the 4073 trainees who began training, there were only 5 symptomatic cases reported, owing to the early implementation of nonpharmaceutical interventions, such as social distancing, rapid isolation, universal masking, recruiting trainees from communities with low COVID-19 prevalence, and a 14-day arrival quarantine.^20^ As the pandemic progressed, operations expanded, with trainees arriving from all parts of the US, including communities with high transmission of COVID-19.

This study is a cohort study examining basic military trainees in a congregant setting with universal entry screening to determine risk factors for subsequent outbreak. We sought to determine whether there is an association between symptoms, laboratory results, and subsequent outbreak in young, healthy individuals who are following recommended public health measures.

## Methods

### Patient Selection

Basic trainees who were diagnosed with COVID-19 by RT-PCR from nasopharyngeal specimens between May 11, 2020, and August 24, 2020, at Joint Base San Antonio–Lackland were enrolled in this study. On arrival, trainees were divided into cohorts of 30 to 50 trainees by sex. Trainees were tested within 48 hours of arrival to basic military training as well as at the end of the 2-week arrival quarantine, regardless of the presence of symptoms. In addition, at any time throughout training, trainees who developed symptoms compatible with COVID-19 were tested at clinician discretion. In cohorts with greater than 30% of trainees with COVID-19, subsequent universal mass testing of the cohorts with RT-PCR was performed. Samples collected for prequarantine and postquarantine testing, as well as those collected for symptoms in the basic training environment, were preferentially tested with the SARS-CoV-2 Real-Time Polymerase Chain Reaction Assay on the Panther Fusion system (Hologic Inc), which was granted emergency use authorization by the US Food and Drug Administration.^21^ If the patient presented to the emergency department or urgent care setting, diagnosis of COVID-19 was based on a positive RT-PCR result in accordance with the platform used, with the platform depending on laboratory testing requirements at collection. In addition, there were times of increased testing demand, in which scheduled quarantine testing also occurred with a platform other than Panther Fusion. All trainees with COVID-19 were immediately isolated for 10 days, regardless of symptoms, and could not return until they were afebrile for 24 hours while not receiving antipyretics. For the purposes of this study, a COVID-19 cluster was defined as 5 cases in a cohort, based on initial signals in these cohorts that there were typically fewer than 4 cases per cohort on initial entry testing, indicative of infection before arrival for training. A contact was defined as having prolonged COVID-19 exposure on contact investigation, including with someone in the same cohort. The 59th Medical Wing institutional review board determined the protocol was public health surveillance, and a waiver was given for informed consent. This article was written in accordance with the Strengthening the Reporting of Observational Studies in Epidemiology (STROBE) reporting guideline.^22^

### Statistical Analysis

Trainees with COVID-19 were interviewed by a clinician at the positive test result, when they were given a standardized form to determine cohort, week of training, symptoms present (including headache, chest pain, fever, chills, cough, dyspnea, congestion, sore throat, runny nose, nausea, vomiting, diarrhea, and anosmia), whether the trainee had a known COVID-19 exposure, and date of symptom onset. All trainees who had their nasopharyngeal swab tested on the Panther Fusion system had the Ct value collected, and those who had testing performed on a different testing platform were recorded as missing so that comparisons were made on only 1 platform.

Categorical variables were compared by χ^2^ or Fisher exact test as appropriate. Continuous variables were compared by Mann-Whitney *U* test. To evaluate Ct value as a marker of symptomatic disease in this cohort, the Ct of asymptomatic trainees was compared with that of symptomatic trainees. Additional comparisons were made for sex, contacts, and individual symptoms. The Ct value for days of symptoms and total number of symptoms reported on screening were compared by a Kruskal-Wallis test.

Cluster cohorts were compared with cohorts without clustered transmission by sex, Ct value, number and type of symptoms present, and days of symptoms. In addition, to identify characteristics that differentiate cohorts on the first day of diagnosed cases on the potential to have an outbreak within the cohort, trainees with COVID-19 on the first day of diagnosis in their cohorts were compared by Ct value, number and type of symptoms present, and days of symptoms. Because individual symptoms could be collinear, a binomial logistic regression was run on the 6 variables found to be significant on univariate analysis, with the exception of chills because it was collinear with fever by Spearman test. Statistical analysis was performed with SPSS statistical software version 22 (IBM Corp). A 2-tailed *P* < .05 was defined as significant for statistical purposes.

## Results

A total of 10 617 trainees entered basic military training during the study period, with 407 trainees (4%; 3830 per 100 000) diagnosed with COVID-19. All trainees were treated as outpatients with supportive care. Of these trainees, 403 (99%) had information on which cohort they were training in and were included for analysis. The median (interquartile range [IQR]) age for this cohort was 20 (19-23) years, and 318 (79%) were men; 204 (51%) were symptomatic, and 199 (49%) were asymptomatic. A total of 260 cases (65%) occurred during arrival quarantine, and 143 (35%) were discovered later in training; 134 cases (52%) were detected on arrival testing, and 85 new cases (33%) were found during asymptomatic testing during week 2. There were 41 trainees (16%) who were identified by symptoms in arrival quarantine.

A total of 336 samples (83%) were run on the Panther Fusion platform and included for comparison on Ct values (Table 1). An additional 44 trainees (11%) tested positive on the BioFire FilmArray COVID-19 test (Biomerieux, Inc), and 23 (6%) tested positive on the GeneXpert Xpress SARS-CoV-2 test (Cepheid, Inc) and were not included in analysis. Lower Ct values were observed in men compared with women (median [IQR], 27.9 [20.2-35.1] vs 33.5 [25.3-37.9]; *P* = .001) and in trainees who were symptomatic compared with those who were asymptomatic (median [IQR], 21.2 [18.4-27.6] vs 34.8 [29.3-37.4]; *P* < .001). Median (IQR) Ct values were also significantly lower in trainees who were in cohorts with clusters of others with COVID-19 infection (25.0 [19.7-31.1] vs 35.4 [25.6-37.8]; *P* = .001) as well as if they had known contact with a person with COVID-19 (30.9 [25.5-35.1] vs 36.3 [31.2-38.0]; *P* < .001). Trainees with known recent contacts had lower Ct values when all trainees and asymptomatic trainees were compared but similar Ct values when only symptomatic trainees were compared. The number of symptoms was inversely associated with Ct values (χ^2^_8_ = 108.9; *P* < .001), and the number of days of symptoms before testing was associated with increased Ct value with time (χ^2^_13_ = 27.861; *P* = .009).

**Table 1.** Reverse Transcription–Polymerase Chain Reaction Cycle Threshold Value for 403 Patients With Coronavirus Disease 2019

Characteristic	Patients, No. (%)	Cycle threshold value, median (IQR)	Missing, No. (%)	*P* value
Women	85 (21)	33.5 (25.3-37.9)	7 (8)	.001
Men	318 (79)	27.9 (20.2-35.1)	60 (19)	
Known contact^a^				
Yes	205 (51)	25.5 (19.95-31.6)	41 (20)	<.001
No	196 (49)	34.4 (22.3-37.6)	26 (13)	
Asymptomatic^b^				
Known contact	74 (37)	30.9 (25.5-35.1)	3 (4)	<.001
No known contact	128 (63)	36.3 (31.2-38.0)	18 (14)	
Symptomatic				
Known contact	131 (66)	21.2 (18.4-26.6)	38 (29)	.52
No known contact	68 (34)	21.1 (18.3-33.1)	8 (12)	
Symptoms				
Asymptomatic	204 (51)	34.8 (29.3-37.4)	21 (10)	<.001
Symptomatic	199 (49)	21.2 (18.4-27.6)	46 (23)	
Chest pain	19 (10)	20.4 (17-24.5)	8 (42)	NA
Dyspnea	37 (19)	20.8 (18.1-26.9)	5 (14)	NA
Cough	69 (34)	19.5 (17.7-26.6)	18 (26)	NA
Fever	45 (22)	19.0 (17.5-22.6)	14 (31)	NA
Headache	101 (51)	20.7 (18.2-26.2)	31 (31)	NA
Chills	46 (23)	20.0 (18.2-22.3)	16 (35)	NA
Nausea	17 (9)	22.3 (20.1-32.2)	4 (24)	NA
Vomiting	3 (2)	NA	3 (100)	NA
Diarrhea	12 (6)	23.9 (20.1-27.4)	4 (33)	NA
Myalgia	73 (37)	20.7 (18.1-24.9)	25 (34)	NA
Sore throat	67 (34)	21.2 (18.6-25.7)	16 (24)	NA
Anosmia	38 (19)	22.2 (19.9-29.6)	7 (18)	NA
Runny nose	31 (16)	22.0 (18.5-29.9)	5 (16)	NA
Congestion	27 (14)	21.2 (18.2-34.3)	3 (11)	NA
Symptoms, No.				
0	204 (51)	34.8 (29.3-37.7)	21 (10)	<.001
1	58 (14)	25.9 (19.3-32.7)	4 (7)	
2	44 (11)	21.1 (18.3-25.3)	11 (25)	
3	34 (8)	18.6 (17.7-22.6)	13 (38)	
4	24 (6)	20.0 (18.4-29.9)	10 (42)	
5	13 (3)	21.3 (17.6-26.5)	1 (8)	
6	12 (3)	20.1 (14.9-22.3)	3 (25)	
7	8 (2)	22.2 (18.6-27.1)	1 (13)	
8	6 (1)	20.4 (19.9-21.3)	3 (50)	
Duration of symptoms, d^c^				
0	30 (15)	21.9 (18.6-27.7)	6 (20)	.009
1	57 (29)	20.3 (17.7-25.5)	19 (33)	
2	37 (19)	19.7 (18.0-22.9)	6 (16)	
3	17 (4)	27.0 (22.7-30.9)	9 (53)	
4	14 (3)	19.3 (17.8-21.1)	3 (21)	
5	5 (2)	21.2 (21.2-23.5)	0	
6	2 (1)	20.1	1 (50)	
7	2 (1)	29.8 (27.6-31.9)	0	
8	1 (<1)	35.1	0	
11	1 (<1)	25.6	0	
14	2 (1)	26.7 (20.0-33.4)	0	
31	4 (1)	37.1 (29.9-38.7)	0	
32	1 (<1)	34.8	0	
35	1 (<1)	23.7	0	

Abbreviations: IQR, interquartile range; NA, not available.

^a^ From 401 of 403 available observations (99.5%).

^b^ From 202 of 204 available observations (99%).

^c^ From 174 of 199 available observations (87%).

Trainees were diagnosed with COVID-19 in 129 of 263 cohorts (49%), of which 14 cohorts (5%) met the criteria for cluster (eTable 1 in the Supplement). Of these cohorts, 11 had individuals with COVID-19 detected at least 5 days after the first case within the cohort, with 1 cohort with others receiving a diagnosis after 4 days, and 1 after 3 days (eTable 2 in the Supplement). One cohort had all cases detected on a single day; this cohort’s Ct values were excluded from analysis because they were missing data. Eleven clusters (79%) occurred in exclusively male cohorts, and 3 (21%) were in exclusively female cohorts. Cohorts with clusters of individuals with COVID-19 infection had a median (IQR) of 12 (9.3-25.3) cases, and cohorts without clusters had a median (IQR) of 1 (1-2) case. Cohorts with COVID-19 clusters had cases occurring later in training compared with cohorts without clusters (median [IQR], week 4 [2-5] vs week 0 [0-2]; *P* < .001) (Table 2). There was no significant difference in median days of symptoms before presentation in cohorts, depending on their cluster status (median [IQR], 2 [1-3] vs 1 [0-3]; *P* = .37). A higher percentage of trainees in cohorts with clusters had symptomatic disease compared with those in cohorts without clusters (146 cohorts [64%] vs 53 cohorts [30%]; *P* < .001).

**Table 2.** Clinical and Laboratory Findings of Patients With COVID-19

Characteristic	No. (%)		*P* value
	Cohorts with COVID-19 clusters (n = 228)	Cohorts without COVID-19 clusters (n = 175)	
Men	204 (89)	114 (64)	<.001
Women	24 (11)	61 (35)	
Time in training, median (IQR), wk	4 (2-5)	0 (0-2)	<.001
Time symptomatic before presentation, median (IQR), d	2 (1-3)	1 (0-3)	.37
Ct, median (IQR)			<.001
Overall	25.0 (19.7-31.1)^a^	35.4 (25.6-37.8)^b^	
Symptomatic patients	21.1 (18.4-26.6)^c^	22.9 (18.3-34.4)^d^	.36
Asymptomatic patients	30.1 (25.9-32.8)^e^	36.5 (32.6-38.0)^f^	<.001
Symptomatic patients	146 (64)	53 (30)	<.001
Symptoms			
Chest pain	13 (9)	6 (11)	.60
Dyspnea	33 (23)	4 (8)	.02
Cough	56 (38)	13 (25)	.07
Headache	82 (56)	19 (36)	.01
Fever	40 (27)	5 (9)	.01
Chills	40 (27)	6 (11)	.02
Nausea	11 (8)	6 (11)	.40
Vomiting	2 (12)	1 (2)	.79
Diarrhea	11 (8)	1 (2)	.14
Myalgia	67 (46)	6 (11)	<.001
Sore throat	53 (36)	14 (26)	.19
Anosmia	33 (23)	5 (8)	.03
Runny nose	28 (19)	3 (6)	.02
Congestion	14 (10)	13 (25)	.01
Symptoms, No.			
0	82 (36)	122 (70)	<.001
1	35 (15)	23 (13)	
2	26 (11)	18 (10)	
3	29 (13)	5 (3)	
4	18 (8)	6 (3)	
5	12 (5)	1 (1)	
6	12 (5)	0	
7	8 (4)	0	
8	6 (3)	0	

Abbreviations: COVID-19, coronavirus disease 2019; Ct, cycle threshold; IQR, interquartile range.

^a^ Based on 178 of 228 available observations (78%).

^b^ Based on 158 of 175 available observations (90%).

^c^ Based on 108 of 146 available observations (74%).

^d^ Based on 45 of 53 available observations (85%).

^e^ Based on 70 of 82 available observations (85%).

^f^ Based on 113 of 122 (93%) available observations.

Based on an analysis of index cases within a cohort with trainees who were diagnosed with COVID-19, there were differences between cohorts that subsequently had a cluster of others with COVID-19 compared with those that did not (Table 3). In cohorts with clusters, 31 of 58 trainees (53%) were symptomatic compared with 43 of 151 trainees (28%) in cohorts that did not subsequently have a cluster. Only 1 cohort (7%) with exclusively asymptomatic trainees had a subsequent cluster. There was no difference in median (IQR) number of days of symptoms before presentation (1 [0-3] days vs 1 [0-4] days; *P* = .49). Furthermore, there was no difference in risk of a development of a cluster if the first patient with COVID-19 presented with symptoms on the first day vs a later day (7 of 31 [23%] vs 11 of 43 [26%]; *P* = .77). However, there was an increased median (IQR) number of symptoms present per patient (3 [2-5] vs 2 [1-2]; *P* = .001) and lower median (IQR) Ct values in trainees in a cohort that subsequently developed a cluster (22.3 [18.4-27.3] vs 35.3 [26.5-37.8]; *P* < .001).

**Table 3.** Clinical and Laboratory Findings of Patients With Coronavirus Disease 2019 Who Presented as the Index Case in Their Cohort

Characteristic	No. (%)		*P* value
	Cluster (n = 58)	No cluster (n = 151)	
Men	47 (81)	100 (66)	.04
Women	11 (19)	51 (34)	
Time symptomatic before presentation, median (IQR), d	1 (0-2)	1 (0-4)	.49
Ct, median (IQR)			<.001
Overall	22.3 (18.4-27.3)^a^	35.3 (26.5-37.8)^b^	
Symptomatic patients	20 (17.8-22.6)^c^	24.35 (18.4-34.4)^d^	.03
Asymptomatic patients	27.2 (23.9-30.2)^e^	36.4 (32.4-38.0)^f^	<.001
Symptomatic patients	31 (53)	43 (28)	.001
Symptoms			
Chest pain	0	6 (14)	.03
Dyspnea	5 (16)	4 (9)	.47
Cough	12 (38)	9 (21)	.09
Headache	20 (65)	13 (30)	.003
Fever	10 (32)	4 (9)	.01
Chills	11 (35)	4 (9)	.01
Nausea	1 (3)	6 (14)	.22
Vomiting	0	1 (2)	NA
Diarrhea	3 (10)	0	.07
Myalgia	11 (35)	4 (9)	.01
Sore throat	14 (45)	10 (23)	.05
Anosmia	7 (23)	4 (9)	.11
Runny nose	9 (29)	3 (7)	.01
Congestion	3 (10)	13 (30)	.03
Symptoms, No.			
0	27 (47)	108 (72)^g^	<.001
1	7 (12)	21 (14)	
2	5 (9)	12 (8)	
3	7 (12)	5 (3)	
4	3 (5)	4 (3)	
5	3 (5)	1 (1)	
6	3 (5)	0	
7	1 (2)	0	
8	2 (3)	0	

Abbreviations: Ct, cycle threshold; IQR, interquartile range; NA, not applicable.

^a^ From 43 of 58 available observations (74%).

^b^ From 140 of 151 available observations (93%).

^c^ From 26 of 31 available observations (84%).

^d^ From 38 of 43 available observations (88%).

^e^ From 17 of 27 available observations (63%).

^f^ From 102 of 108 available observations (94%).

^g^ Percentages may not add up to 100% owing to rounding.

In univariate analysis, reported symptoms of headache, fever, chills, myalgia, and runny nose were more common in cohorts that subsequently developed clusters of 5 or more individuals with COVID-19 infection, whereas chest pain and congestion were more common in cohorts that did not develop clusters. However, in multivariate analysis, only congestion (odds ratio, 0.2; 95% CI, 0.06-0.6; *P* = .02) remained significant and was negatively associated with clustered cohorts, but the number of trainees with this symptom was small (eTable 3 in the Supplement).

## Discussion

This study was designed to assess symptoms and laboratory values of a young, healthy population living in congregant-setting cohorts, with consistent and extensive use of nonpharmaceutical interventions (such as physical distancing and rapid isolation of symptomatic patients), who were exposed to individuals with COVID-19 infection, and to assess factors associated with subsequent development of clusters of cases of others with COVID-19 infection within these cohorts. With 10 617 entering trainees and 403 (3%) receiving a diagnosis of COVID-19 infection, this analysis includes among the largest series of clustered transmission of COVID-19 infection in a controlled environment. The success of the US Air Force basic military training response can provide insight into interventions that may prevent transmission in other congregant settings.

One finding of this study was the apparent efficacy of nonpharmaceutical interventions in preventing outbreaks among the trainees. The basic military training population has a much lower attack rate compared with those described earlier in the pandemic, when infection control measures were not fully implemented.^23,24^ Although trainees presented with COVID-19 in almost half of all cohorts, only 11% of cohorts that had 1 individual infected with COVID-19 subsequently developed a cluster of 5 or more cases. Although the investment in nonpharmaceutical interventions was significant, it allowed for the continuation of the training mission during times with significant community spread outside of basic military training, including the San Antonio community around Joint Base San Antonio–Lackland.^25^ Although the basic trainees have no direct access to the local community, the training staff are not restricted to the military base and may be a source of SARS-CoV-2 transmission to the basic military training population. Implementation of nonpharmaceutical interventions will continue to be central to any response that involves congregant settings with a high turnover and regular importation of disease from community settings.

An additional finding is that Ct, as a surrogate of viral load at the sampling site, may be useful in evaluating patients in a congregant setting, even in patients with mild disease. Both recent acquisition of disease (as demonstrated by trainees with known contacts) and ongoing transmission (as observed in cohorts with clusters of other individuals infected with COVID-19) were associated with lower Ct values. In addition, trainees with an increased number of symptoms had lower Ct values. The lower Ct value in men may be associated with more symptomatic cases and greater likelihood of being in a cluster cohort, for reasons that could include nonpharmaceutical intervention adherence or biological differences in disease course based on sex.^26^ No individual symptom was associated with significantly different Ct values. This is consistent with data showing that symptoms do not have any difference in length of RT-PCR positivity.^27^ Taken together, these findings suggest that Ct values may be useful in assessing risk of ongoing transmission among specific cohorts, and there may be benefit in comparing Ct values of different cohorts to understand transmission potential if the findings are validated in future studies.

There are many case series suggestive of asymptomatic spread in congregant settings.^28,29,30^ Early modeling studies have also shown that presymptomatic transmission has been a driver of spread.^31^ However, more recent criticisms of these studies have shown that modeling studies overestimated the contribution of asymptomatic transmission compared with epidemiologic data.^32^ This study suggests that asymptomatic trainees made only a modest contribution to transmission, in line with other epidemiologic studies.^33^ Only 1 of 14 cohorts with clustered cases had no symptomatic cases on the first day of index case diagnosis. This finding suggests that asymptomatic transmission may not have been a major contributor to outbreaks in this controlled setting with high nonpharmaceutical intervention implementation.

One hypothesis for superspreader events is that symptomatic individuals who do not seek health care may cause widespread dissemination of disease. This has been shown in other high-transmission events in the early days of the pandemic, in which transmission reached 45% of subsequent cases of infected individuals.^34,35^ In the setting of this study, with rapid isolation of trainees infected with COVID-19, it is surprising that whether a cohort subsequently developed a cluster of 5 or more individuals infected with COVID-19 was not associated with days of symptoms of the index cases. A possible explanation is that subsequent transmission was associated with other factors, such as viral load or types of symptoms. Several symptoms were significantly associated with development of clustered cases in univariate analysis; however, in multivariate analysis, only congestion was significantly negatively associated with subsequent outbreaks in cohorts, and the number of trainees with this symptom was small.

It has been shown that rapid identification of symptomatic individuals over population-level measures was the most effective at eliminating superspreaders in prior outbreaks.^36^ In our study, trainees with more symptoms and lower Ct levels had the highest risk of starting disease clusters, even in this setting with rapid isolation. One promising tool, viral antigen testing, has the potential to rapidly identify these individuals because these tests perform with better sensitivity as viral loads increase and may be better at identifying patients with a higher chance of transmission.^37^ Further work is needed to elucidate the role of antigen tests in the COVID-19 response.

### Limitations

There are several limitations to this study. This analysis was performed in a cohort of healthy young trainees who were prescreened for underlying medical conditions, and its generalizability to other populations is unclear. The aggressive nonpharmaceutical intervention measures used in this study may have been associated with more protection from asymptomatic transmission. The definition used for cluster may not fully encapsulate transmission because not all cohorts with greater than 5 cases may have been the result of person-to-person transmission, and there may have been limited person-to-person transmission with numbers of cases not meeting the cluster definition used here. This study did not rule out asymptomatic transmission in the days preceding transmission, which may have been underreported. Furthermore, conclusions on transmission dynamics are limited in the absence of genetic sequencing. Additionally, Ct values were more commonly missing for symptomatic patients, which may have led to bias in these results. Furthermore, only mild disease was observed in this cohort, and it is unclear whether results would differ in cohorts with more severe disease, such as hospitalized patients.

## Conclusions

In this study of 10 613 US Air Force trainees living in a congregant setting, 403 (3%) received a diagnosis of COVID-19 infection. These findings show the potential effectiveness of continued nonpharmaceutical intervention efforts at containing SARS-CoV-2 in the midst of a worldwide pandemic with continued importation of cases and with hundreds of trainees from high-transmission areas arriving weekly at a congregant setting. In addition, higher numbers of symptoms and lower Ct values were associated with cohorts that subsequently developed clusters of 5 or more individuals with COVID-19 infection. A combination of continued nonpharmaceutical interventions and rapid identification of patients with higher transmission potential may be a key in preventing subsequent larger outbreaks in high-risk settings.

## Notes

Supplement. **eTable 1.** Summary of Demographic Characteristics and COVID-19 Cases in Cohorts With Case Clusters **eTable 2.** Number of Cases per Day for Each Flight That Met the Definition of a Cluster **eTable 3.** Univariate and Binomial Logistic Regression of Symptoms Present on Day 1 of Cases for Index Case in a Cohort and Subsequent Risk of Outbreak

## References

[1] Hagan LM, Williams SP, Spaulding AC, . Mass testing for SARS-CoV-2 in 16 prisons and jails—six jurisdictions, United States, April-May 2020. MMWR Morb Mortal Wkly Rep. 2020;69(33):1139-1143. doi:10.15585/mmwr.mm6933a3 32817597 PMC7439979

[2] Roxby AC, Greninger AL, Hatfield KM, . Outbreak investigation of COVID-19 among residents and staff of an independent and assisted living community for older adults in Seattle, Washington. JAMA Intern Med. 2020;180(8):1101-1105. 32437547 10.1001/jamainternmed.2020.2233 PMC7292007

[3] Payne DC, Smith-Jeffcoat SE, Nowak G, ; CDC COVID-19 Surge Laboratory Group. SARS-CoV-2 infections and serologic responses from a sample of US Navy service members—USS Theodore Roosevelt, April 2020. MMWR Morb Mortal Wkly Rep. 2020;69(23):714-721. doi:10.15585/mmwr.mm6923e4 32525850 PMC7315794

[4] Bodkin C, Mokashi V, Beal K, . Pandemic planning in homeless shelters: a pilot study of a COVID-19 testing and support program to mitigate the risk of COVID-19 outbreaks in congregate settings. Clin Infect Dis. Published online June 8, 2020. doi:10.1093/cid/ciaa743 32511704 PMC7314119

[5] Pan A, Liu L, Wang C, . Association of public health interventions with the epidemiology of the COVID-19 outbreak in Wuhan, China. JAMA. 2020;323(19):1915-1923. doi:10.1001/jama.2020.6130 32275295 PMC7149375

[6] Davlantes E, Toro M, Villalobos R, Sanchez-Gonzalez L. Notes from the field: COVID-19 prevention practices in state prisons—Puerto Rico, 2020. MMWR Morb Mortal Wkly Rep. 2020;69(33):1144. doi:10.15585/mmwr.mm6933a4 32817601 PMC7439978

[7] Szablewski CM, Chang KT, Brown MM, . SARS-CoV-2 transmission and infection among attendees of an overnight camp—Georgia, June 2020. MMWR Morb Mortal Wkly Rep. 2020;69(31):1023-1025. doi:10.15585/mmwr.mm6931e1 32759921 PMC7454898

[8] Luo L, Liu D, Liao X, . Contact settings and risk for transmission in 3410 close contacts of patients with COVID-19 in Guangzhou, China: a prospective cohort study. Ann Intern Med. 2020;173(11):879-887. doi:10.7326/M20-2671 32790510 PMC7506769

[9] Chau NVV, Thanh Lam V, Thanh Dung N, . The natural history and transmission potential of asymptomatic SARS-CoV-2 infection. Clin Infect Dis. 2020;71(10):2679-2687. doi:10.1093/cid/ciaa711 32497212 PMC7314145

[10] Cheng HY, Jian SW, Liu DP, Ng TC, Huang WT, Lin HH; Taiwan COVID-19 Outbreak Investigation Team. Contact tracing assessment of COVID-19 transmission dynamics in Taiwan and risk at different exposure periods before and after symptom onset. JAMA Intern Med. 2020;180(9):1156-1163. doi:10.1001/jamainternmed.2020.2020 32356867 PMC7195694

[11] Huang L, Zhang X, Zhang X, . Rapid asymptomatic transmission of COVID-19 during the incubation period demonstrating strong infectivity in a cluster of youngsters aged 16-23 years outside Wuhan and characteristics of young patients with COVID-19: a prospective contact-tracing study. J Infect. 2020;80(6):e1-e13. doi:10.1016/j.jinf.2020.03.006 32283156 PMC7194554

[12] Hellewell J, Abbott S, Gimma A, ; Centre for the Mathematical Modelling of Infectious Diseases COVID-19 Working Group. Feasibility of controlling COVID-19 outbreaks by isolation of cases and contacts. Lancet Glob Health. 2020;8(4):e488-e496. doi:10.1016/S2214-109X(20)30074-7 32119825 PMC7097845

[13] Liu Y, Yan LM, Wan L, . Viral dynamics in mild and severe cases of COVID-19. Lancet Infect Dis. 2020;20(6):656-657. doi:10.1016/S1473-3099(20)30232-2 32199493 PMC7158902

[14] Magleby R, Westblade LF, Trzebucki A, . Impact of SARS-CoV-2 viral load on risk of intubation and mortality among hospitalized patients with coronavirus disease 2019. Clin Infect Dis. 2020;ciaa851. doi:10.1093/cid/ciaa851 32603425 PMC7337625

[15] Huang CG, Lee KM, Hsiao MJ, . Culture-based virus isolation to evaluate potential infectivity of clinical specimens tested for COVID-19. J Clin Microbiol. 2020;58(8):e01068-20. doi:10.1128/JCM.01068-20 32518072 PMC7383522

[16] Rhoads D, Peaper DR, She RC, . College of American Pathologists (CAP) Microbiology Committee perspective: caution must be used in interpreting the cycle threshold (Ct) value. Clin Infect Dis. Published online August 12, 2020. doi:10.1093/cid/ciaa1199 32785682

[17] Gray GC, Callahan JD, Hawksworth AW, Fisher CA, Gaydos JC. Respiratory diseases among US military personnel: countering emerging threats. Emerg Infect Dis. 1999;5(3):379-385. doi:10.3201/eid0503.990308 10341174 PMC2640764

[18] Tate JE, Bunning ML, Lott L, . Outbreak of severe respiratory disease associated with emergent human adenovirus serotype 14 at a US Air Force training facility in 2007. J Infect Dis. 2009;199(10):1419-1426. doi:10.1086/598520 19351260

[19] Banta J, Lenz B, Pawlak M, . Notes from the field: outbreak of hand, foot, and mouth disease caused by coxsackievirus A6 among basic military trainees—Texas, 2015. MMWR Morb Mortal Wkly Rep. 2016;65(26):678-680. doi:10.15585/mmwr.mm6526a3 27388434

[20] Marcus JE, Frankel DN, Pawlak MT, . COVID-19 monitoring and response among US Air Force basic military trainees—Texas, March-April 2020. MMWR Morb Mortal Wkly Rep. 2020;69(22):685-688. doi:10.15585/mmwr.mm6922e2 32497031 PMC7315849

[21] Hogan CA, Sahoo MK, Huang C, . Comparison of the Panther Fusion and a laboratory-developed test targeting the envelope gene for detection of SARS-CoV-2. J Clin Virol. 2020;127:104383. doi:10.1016/j.jcv.2020.104383 32353760 PMC7195328

[22] von Elm E, Altman DG, Egger M, Pocock SJ, Gøtzsche PC, Vandenbroucke JP; STROBE Initiative. Strengthening the Reporting of Observational Studies in Epidemiology (STROBE) statement: guidelines for reporting observational studies. BMJ. 2007;335(7624):806-808. doi:10.1136/bmj.39335.541782.AD 17947786 PMC2034723

[23] Park SY, Kim YM, Yi S, . Coronavirus disease outbreak in call center, South Korea. Emerg Infect Dis. 2020;26(8):1666-1670. doi:10.3201/eid2608.201274 32324530 PMC7392450

[24] James A, Eagle L, Phillips C, . High COVID-19 attack rate among attendees at events at a church—Arkansas, March 2020. MMWR Morb Mortal Wkly Rep. 2020;69(20):632-635. doi:10.15585/mmwr.mm6920e2 32437338

[25] Selcraig B. San Antonio reports 1,688 new coronavirus cases; mayor blames July 4 gatherings. *San Antonio Express News*. Published July 22, 2020. Accessed September 15, 2020. https://www.expressnews.com/news/local/article/San-Antonio-reports-1-688-new-novel-coronavirus-15427345.php

[26] Jin JM, Bai P, He W, . Gender differences in patients with COVID-19: focus on severity and mortality. Front Public Health. 2020;8:152. doi:10.3389/fpubh.2020.00152 32411652 PMC7201103

[27] Corsini Campioli C, Cano Cevallos E, Assi M, Patel R, Binnicker MJ, O’Horo JC. Clinical predictors and timing of cessation of viral RNA shedding in patients with COVID-19. J Clin Virol. 2020;130:104577. doi:10.1016/j.jcv.2020.104577 32777762 PMC7405830

[28] Mizumoto K, Kagaya K, Zarebski A, Chowell G. Estimating the asymptomatic proportion of coronavirus disease 2019 (COVID-19) cases on board the *Diamond Princess* cruise ship, Yokohama, Japan, 2020. Euro Surveill. 2020;25(10):2000180. doi:10.2807/1560-7917.ES.2020.25.10.2000180 32183930 PMC7078829

[29] Jiang XL, Zhang XL, Zhao XN, . Transmission potential of asymptomatic and paucisymptomatic severe acute respiratory syndrome coronavirus 2 infections: a 3-family cluster study in China. J Infect Dis. 2020;221(12):1948-1952. doi:10.1093/infdis/jiaa206 32319519 PMC7188140

[30] Arons MM, Hatfield KM, Reddy SC, ; Public Health–Seattle and King County and CDC COVID-19 Investigation Team. Presymptomatic SARS-CoV-2 infections and transmission in a skilled nursing facility. N Engl J Med. 2020;382(22):2081-2090. doi:10.1056/NEJMoa2008457 32329971 PMC7200056

[31] He X, Lau EHY, Wu P, . Temporal dynamics in viral shedding and transmissibility of COVID-19. Nat Med. 2020;26(5):672-675. doi:10.1038/s41591-020-0869-5 32296168

[32] Slifka MK, Gao L. Is presymptomatic spread a major contributor to COVID-19 transmission? Nat Med. 2020;26(10):1531-1533. doi:10.1038/s41591-020-1046-6 32807936

[33] Wei WE, Li Z, Chiew CJ, Yong SE, Toh MP, Lee VJ. Presymptomatic transmission of SARS-CoV-2—Singapore, January 23–March 16, 2020. MMWR Morb Mortal Wkly Rep. 2020;69(14):411-415. doi:10.15585/mmwr.mm6914e1 32271722 PMC7147908

[34] Hamner L, Dubbel P, Capron I, . High SARS-CoV-2 attack rate following exposure at a choir practice—Skagit County, Washington, March 2020. MMWR Morb Mortal Wkly Rep. 2020;69(19):606-610. doi:10.15585/mmwr.mm6919e6 32407303

[35] Dyal JW, Grant MP, Broadwater K, . COVID-19 among workers in meat and poultry processing facilities—19 states, April 2020. MMWR Morb Mortal Wkly Rep. 2020;69(18). doi:10.15585/mmwr.mm6918e3 32379731

[36] Lloyd-Smith JO, Schreiber SJ, Kopp PE, Getz WM. Superspreading and the effect of individual variation on disease emergence. Nature. 2005;438(7066):355-359. doi:10.1038/nature04153 16292310 PMC7094981

[37] Hirotsu Y, Maejima M, Shibusawa M, . Comparison of automated SARS-CoV-2 antigen test for COVID-19 infection with quantitative RT-PCR using 313 nasopharyngeal swabs, including from seven serially followed patients. Int J Infect Dis. 2020;99:397-402. doi:10.1016/j.ijid.2020.08.029 32800855 PMC7422837

