# Supplementary material for: Risk Factors Associated With COVID-19 Transmission Among US Air Force Trainees in a Congregant Setting
Source: JAMA Netw Open. 2021 Feb 25;4(2):e210202. doi: 10.1001/jamanetworkopen.2021.0202 (PMC7907953; doi:10.1001/jamanetworkopen.2021.0202)
Supplement: Supplement. — eTable 1. Summary of Demographic Characteristics and COVID-19 Cases in Cohorts With Case Clusters eTable 2. Number of Cases per Day for Each Flight That Met the Definition of a Cluster eTable 3. Univariate and Binomial Logistic Regression of Symptoms Present on Day 1 of Cases for Index Case in a Cohort and Subsequent Risk of Outbreak [file jamanetwopen-e210202-s001.pdf]

Supplemental Online Content

Marcus JE, Frankel DN, Pawlak MT, et al. Risk factors associated with COVID-19 transmission among US Air Force trainees in a congregant setting. *JAMA Netw Open*. 2021;4(2):e210202. doi:10.1001/jamanetworkopen.2021.0202

eTable 1. Summary of Demographic Characteristics and COVID-19 Cases in Cohorts With Case Clusters

eTable 2. Number of Cases per Day for Each Flight That Met the Definition of a Cluster

eTable 3. Univariate and Binomial Logistic Regression of Symptoms Present on Day 1 of Cases for Index Case in a Cohort and Subsequent Risk of Outbreak

This supplemental material has been provided by the authors to give readers additional information about their work.

© 2021 Marcus JE. *JAMA Network Open*.

eTable 1. Summary of Demographic Characteristics and COVID-19 Cases in Cohorts With Case Clusters

Cohort	Gender	Week of Training of first case in cluster	Clustered cases in arrival quarantine	Number of Positive COVID-19 cases	Median cycle threshold on day 1 (95% CI)[a]	Median number of symptoms on day 1(95% CI)	Median cycle threshold for all cases in cluster (95% CI)[a]
1	Female	7	No	6	23.7 (21.6-26.0)	5 (4-5)	25.3 (22.1-28.2)
2	Male	5	No	8	17.7 (16.9-20.0)	1 (1-2)	19.3 (18.3-20.8)
3	Female	6	No	8	18.4 (14.2-27.1)	5 (2-7)	22.4 (15.3-27.0)
4	Male	0	Yes	9	--	0	--
5	Male	7	No	10	--	4 (3-4)	--
6	Female	2	Yes	10	18.1	1	23.8 (20.6-32.3)
7	Male	6	No	12	22.3	6	19.0 (16.1-22.3)
8	Male	5	No	12	13.5	6	17.8 (16.5-18.2)
9	Male	1	Yes	13	19.9	8	31.1 (24.0-32.8)
10	Male	4	No	17	22.6 (17.8-22.6)	3 (2-3)	21.1 (19.9-22.6)
11	Male	2	Yes	28	25.5 (20.1-29.7)	0 (0-3)	23.9 (18.6-28.5)
12	Male	5	No	28	--	2 (1-3)	31.1 (21.8-34.0)
13	Male	4	No	31	19.5	3	20.6 (18-23.7)
14	Male	0	Yes	36	29.2 (27.1-31.4)	0	30.1 (27.0-32.1)

[a] Of cases with available data. In clusters without known cycle threshold values marked by “--"

© 2021 Marcus JE. *JAMA Network Open*.

eTable 2. Number of Cases per Day for Each Flight That Met the Definition of a Cluster

Cohort	Pre-Cluster[a]	Day 1	Day 2	Day 3	Day 4	Day 5	Day 6	Day 7	Day 8	Day 9	Day 10	Day 11	Day 12	Day 13	Day 14	Day 15	Day 16	Day 17
1		4		1	1													
2		3	4	1														
3	1	6											1					
4		9																
5	1	2	1	2		1	3											
6		1						1					7					1
7	1	1			4	2		1	1	2								
8		1		2		1	4	2	2									
9		1	1			1			10									
10	1	3		1	4	3	3	2										
11		20	1	5	1									1				
12		2	3	1	1				20				1					
13		1	6		9	4	6	3	2									
14		4			1		1	1								29		

[a]Defined as a COVID-19 case occurring more than 21 days before day 1.

© 2021 Marcus JE. *JAMA Network Open*.

eTable 3. Univariate and Binomial Logistic Regression of Symptoms Present on Day 1 of Cases for Index Case in a Cohort and Subsequent Risk of Outbreak

**Symptoms on Day 1**	**Cluster (n=58)**	**No Cluster (n=151)**	**p-value**	**Logistic Regression Odds Ratio (95% CI)**	**p-value**
Chest Pain	0	6 (14%)	0.03	0	1
Headache	20 (65%)	13 (30%)	0.003	1.4 (0.5-3.7)	0.46
Fever	10 (32%)	4 (9%)	0.01	1.67 (0.4-1.7)	0.49
Myalgia	11 (35%)	4 (9%)	0.01	2.4 (0.5-11)	0.28
Runny Nose	9 (29%)	3 (7%)	0.01	1.7 (0.4-7)	0.47
Congestion	3 (10%)	13 (30%)	0.03	0.2 (.06-.6)	0.02

© 2021 Marcus JE. *JAMA Network Open*.
